# Harnessing Activin A Adjuvanticity to Promote Antibody Responses to BG505 HIV Envelope Trimers

**DOI:** 10.3389/fimmu.2020.01213

**Published:** 2020-06-16

**Authors:** Diane G. Carnathan, Kirti Kaushik, Ali H. Ellebedy, Chiamaka A. Enemuo, Etse H. Gebru, Pallavi Dhadvai, Mohammed Ata Ur Rasheed, Matthias G. Pauthner, Gabriel Ozorowski, Rafi Ahmed, Dennis R. Burton, Andrew B. Ward, Guido Silvestri, Shane Crotty, Michela Locci

**Affiliations:** ^1^Yerkes National Primate Research Center, Emory University, Atlanta, GA, United States; ^2^Scripps Center for HIV/AIDS Vaccine Immunogen Development (CHAVD), The Scripps Research Institute, La Jolla, CA, United States; ^3^Emory Vaccine Center, School of Medicine, Emory University, Atlanta, GA, United States; ^4^Center for Infectious Disease and Vaccine Research, La Jolla Institute for Immunology (LJI), La Jolla, CA, United States; ^5^Department of Microbiology and Immunology, School of Medicine, Emory University, Atlanta, GA, United States; ^6^Department of Pathology and Immunology, Washington University School of Medicine, St. Louis, MO, United States; ^7^Department of Immunology and Microbiology, The Scripps Research Institute, La Jolla, CA, United States; ^8^Department of Integrative Structural and Computational Biology, The Scripps Research Institute, La Jolla, CA, United States; ^9^Ragon Institute of MGH, MIT and Harvard, Cambridge, MA, United States; ^10^Division of Infectious Diseases and Global Public Health, Department of Medicine, University of California, San Diego, La Jolla, CA, United States; ^11^Department of Microbiology, Perelman School of Medicine, University of Pennsylvania, Philadelphia, PA, United States

**Keywords:** HIV, vaccine, T follicular helper cells, T follicular regulatory cells, antibody longevity, B cells

## Abstract

T follicular helper (T_FH_) cells are powerful regulators of affinity matured long-lived plasma cells. Eliciting protective, long-lasting antibody responses to achieve persistent immunity is the goal of most successful vaccines. Thus, there is potential in manipulating T_FH_ cell responses. Herein, we describe an HIV vaccine development approach exploiting the cytokine activin A to improve antibody responses against recombinant HIV Envelope (Env) trimers in non-human primates. Administration of activin A improved the magnitude of Env-specific antibodies over time and promoted a significant increase in Env-specific plasma cells in the bone marrow. The boost in antibody responses was associated with reduced frequencies of T follicular regulatory (T_FR_) cells and increased germinal center T follicular helper (GC-T_FH_) to T_FR_ cell ratios. Overall, these findings suggest that adjuvants inducing activin A production could potentially be incorporated in future rational design vaccine strategies aimed at improving germinal centers, long-lived plasma cells, and sustained antibody responses.

## Introduction

Over 30 million people are currently living with HIV, and developing a protective vaccine for HIV is still a global health priority (1). The discovery that a fraction of HIV-infected individuals can produce antibodies (Abs) capable of neutralizing the majority of HIV circulating strains in *in vitro* neutralization assays and *in vivo* passive transfer experiments has revolutionized the rational design of vaccines for HIV (2–4). Indeed, it is now believed that a vaccine capable of eliciting such broadly neutralizing Abs (bnAbs) could effectively protect vaccinated individuals from HIV infection. The goal of generating bnAbs by immunization is an unprecedented challenge due to many reasons, including the high level of somatic hypermutation present in most bnAbs and the immunodominance of non-neutralizing epitopes in HIV envelope trimers (2, 5). To circumvent these obstacles, multiple approaches aimed at focusing B cell responses on neutralizing epitopes and fostering somatic hypermutation will likely be required (3, 6). An additional issue associated with rational design of vaccines for HIV is the durability of neutralizing Abs (nAbs) elicited by protein immunizations. In non-human primate (NHP) studies, immunization with BG505 SOSIP, an immunogen mimicking native HIV envelope (Env) trimer, can lead to the generation of high nAb titers protecting from subsequent infections with simian-human immunodeficiency virus (SHIV) (7). Nevertheless, the finding that this protection is lost as nAbs progressively wane over time (7) highlights the need for identifying approaches to improve the longevity of vaccine-elicited nAbs.

Serological memory is maintained for decades without antigen re-exposure by long-lived plasma cells (LLPC) residing in the bone marrow (8). High affinity LLPC are formed during the germinal center (GC) reaction, a process where somatic hypermutation is followed by positive selection of high affinity GC B cells (9). The GC reaction, which is the foundation of affinity maturation, is strictly regulated by a subset of CD4 T cells named T follicular helper (T_FH_) cells. T_FH_ cells are necessary for GC formation as well as for the generation of affinity matured LLPC (10, 11).

The differentiation of T_FH_ cells is a complex multifactorial process (10, 11). During this process, distinct costimulatory and cytokine-mediated signals provided by dendritic cells and B cells integrate to coordinate a unique gene program controlling the homing and the B cell helper properties of T_FH_ cells. We recently identified the cytokine activin A as potent inducer of human T_FH_ cell differentiation (12). Activin A, a homodimer of the inhibin beta A protein, is a pleiotropic cytokine regulating many crucial biological processes, including wound healing and stem cell pluripotency (13–15). This cytokine can be promptly produced by professional antigen presenting cells, such as dendritic cells, upon stimulation with TLR agonists or co-stimulatory molecules (12, 15). Type I and II receptors for activin A are expressed by a variety of immune system cells, including naïve T cells (12), and binding of these receptors by activin A results in activation of the SMAD2/3 pathway and downstream regulation of target gene expression (12, 13). We have previously shown that, *in vitro*, activin A shapes multiple facets of T_FH_ biology by modulating the expression of molecules that are important for T_FH_ cell localization (CCR7, CXCR5), induction of the T_FH_ gene program (BCL6, PRDM1), homeostasis (PD-1) and function (CXCL13, TNF) (12). Hence, activin A might be an appealing target to fine-tune Ab responses *in vivo* during vaccination via modulation of T_FH_ cells. Herein, we report our attempt to modulate T_FH_ cell and Ab responses during immunization of rhesus macaques (RM) with BG505 SOSIP Env trimer.

## Materials and Methods

### Animals

Twelve outbred male Indian RMs (*Macaca mulatta*) between 3 and 4 years of age were housed at the Yerkes National Primate Research Center and maintained in accordance with NIH guidelines. This study was approved by the Emory University Institutional Animal Care and Use Committee (IACUC). All animals were treated with anesthesia (ketamine) and analgesics for procedures as per veterinarian recommendations and IACUC approved protocol. Animals were grouped to divide age and weight as evenly as possible (Supplementary Table 1).

### Immunizations and Treatment

All animals were immunized two times, 2 months apart (week 0 and week 8). Subcutaneous immunizations were administered divided between right and left mid-thighs. For each immunization site, 50 μg of BG505 SOSIPv5.2 mixed with 30 U of ISCOMATRIX (CSL Limited) were injected in each leg for a total of 100 μg of antigen and 60 U of adjuvant. Recombinant, carrier-free human/mouse/rat activin A produced in a Chinese Hamster Ovary cell line (R&D Systems) was previously shown to have biological activity on rhesus monkeys (12). Lyophilized activin A was dissolved in PBS and injected in 500 μl/leg. Half the animals were given activin A at 50 μg/kg daily for 3 days beginning on the day of immunization. The dose was split between legs via subcutaneous injection close to the inguinal area but not in the inguinal fold. Animals were euthanized at 14 weeks after the start of the immunization series.

### Lymph Node (LN) Processing and Blood Collection

Iliac LNs were collected at the necropsy time point (week 14). Iliac LNs were grouped as “right” and “left” samples and analyzed independently. The samples were dissociated through 70 μM strainers and washed with PBS. Blood was collected at various time points using serum collection tubes and serum samples were subsequently frozen.

### BG505 Native-Like Env Trimer Immunogens

BG505 SOSIP.v5.2 were generated by Dr. Ward's group. The experimental procedure has been previously described in detail (16). BG505 SOSIP.v5.2 trimers were expressed in HEK293F cells by transient co-transfection with furin. The BG505 SOSIP.v5.2 trimer builds upon the v4.1 design, with the addition of a second disulfide bond (A73C-A561C) between gp120 and gp41 to further increase trimer stability (17). The proteins were purified using PGT145-affinity columns followed by SEC. These proteins had no His-tag (terminal residue D664 of gp41). Fractions corresponding to trimer were pooled and concentrated down to ~0.8 mg/ml in Tris-buffered saline (50 mM Tris pH 7.4, 150 mM NaCl). Structural validation of trimers was performed by analyzing negative-stain electron microscopy (EM) 2D class averages. All samples were filter sterilized prior to aliquoting and flash freezing.

### Flow Cytometry

Multi-color flow cytometric analysis was performed on mononuclear cells isolated from iliac LN samples. The following antibodies were used: LIVE/DEAD dead cell stain kit (Invitrogen); anti-CD8a (clone RPA-T8), anti-CD4 (clone OKT4), anti-PD-1 (clone EH12.2H7), anti-ICOS (clone C398.4A), anti-CD25 (clone BC96), anti-CXCR3 (clone G025H7) (BioLegend); anti-CXCR5 (clone MU5UBEE), anti-FOXP3 (eBioscience); anti-Bcl-6 (clone K112-91), anti-CD95 (clone DX2), anti-CD3 (clone SP34-2), and anti-Ki-67 (clone B56) (BD Biosciences); and anti-CD20 (clone B9E9), IgG (clone G18-145), IgM (G20-127) (Beckman Coulter).

For each BG505 SOSIPv5.2 Env trimer probe analysis, the biotinylated probes were individually premixed with fluorochrome-streptavidin conjugates (SA-Alexa647 and SA-BV421, Thermo Fisher Scientific and BioLegend) at room temperature (RT) for 20 min. After surface staining followed by washes, cells were fixed and permeabilized using FoxP3/Transcription Factor Staining Buffer kit (Thermo Fisher Scientific) according to manufacturer's protocols. Upon permeabilization, cells were stained with intranuclear Abs, washed twice and acquired on an LSR Fortessa Cell Analyzer (BD Biosciences). Flow cytometry data were analyzed with FlowJo (Tree Star).

### BG505 Env Trimer and Env-V3-Loop ELISA

The detailed protocol of BG505 Env trimer ELISA was previously described (16). Endpoint titers were calculated as dilution at which O.D. signal was 0.1 above background using GraphPad Prism.

V3-peptdide ELISA assays weres performed exactly as BG505 Env trimer ELISAs, with the following modification: BG505 V3-peptides (TRPNNNTRKSIRIGPGQAFYATG) were directly coated to 96-well plates at 2.5 μg/mL in PBS overnight.

### ELISPOT for the Detection of Env Trimer-Specific Ab Secreting Cells

ELISPOT for the detection of Env trimer-specific Ab secreting cells has been previously described (18).

### Pseudovirus Neutralization Assays

A detailed description of the neutralization assays was previously published (16). Neutralizing titers were measured in 3 independent experiments and average neutralization titers were calculated.

### Statistical Analysis

Graphpad Prism v7.0 or 8.0 was used for all statistical analyses. Significance of differences were calculated using unpaired, two-tailed Mann–Whitney tests.

## Results

**Figure 1:**
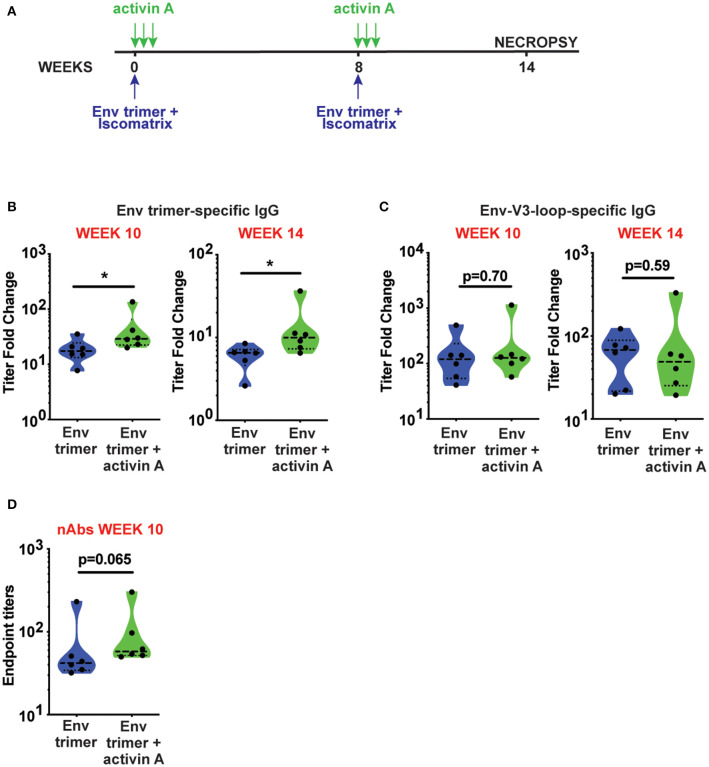
Activin A modulated generation of BG505 SOSIP Env trimer-specific Abs. **(A)** Timeline of immunizations (blue) and activin A administration (green). **(B,C)** Env trimer **(B)** and Env-V3-loop **(C)**-specific IgG titers at week 10 and week 14 are shown as fold change in titers [ratio between Env-specific ELISA endpoint titers at week 10 or week 14 and the pre-immune titers (week-1)]. **(D)** Neutralizing Ab (nAb) titers were measured at week 10 post immunization. Limit of detection of the neutralization assay is 1:10. In **(B–D)**, violin plots show median and quartiles. Each symbol represents an individual animal *p < 0.05.

**Figure 2:**
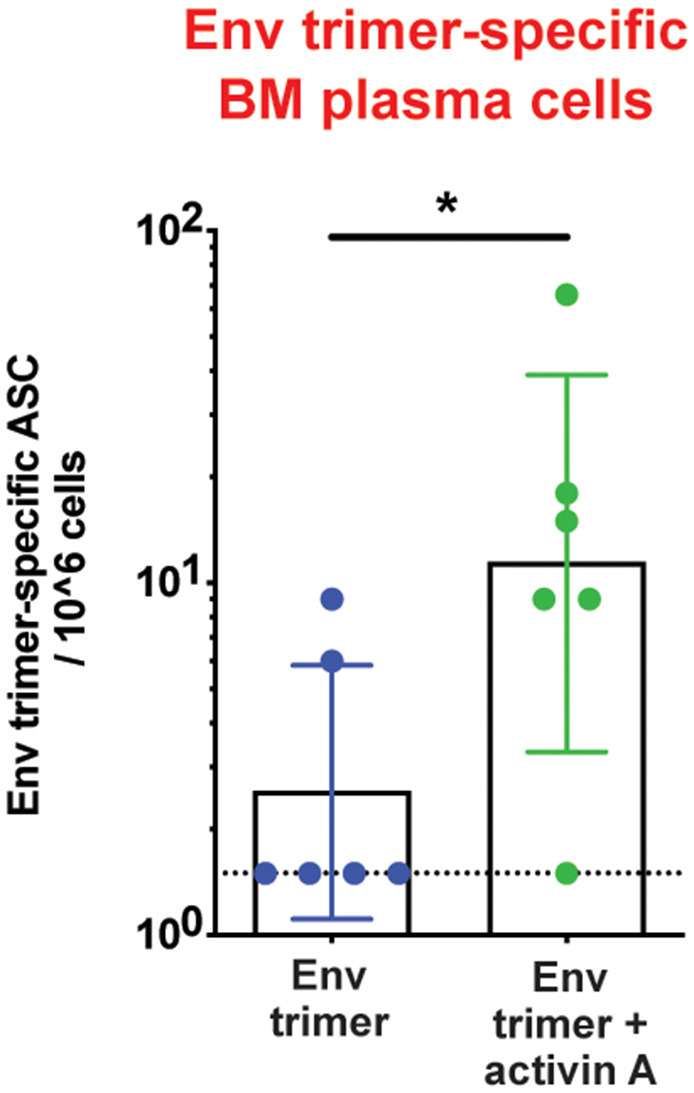
Activin A's regulation of bone marrow Ab-secreting cells. Bone marrow Env trimer-specific IgG secreting cells were measured by ELISPOT at the necropsy time point. Bars show geometric mean with geometric SD. Dotted line represents limit of detection. *p < 0.05.

To assess the adjuvanticity of activin A and its ability to foster T_FH_ cells and Ab responses *in vivo*, we designed a study where 12 rhesus monkeys (RM) were immunized with BG505 SOSIPv5.2 HIV Env trimer protein formulated with an ISCOM-class saponin adjuvant (ISCOMATRIX). All the animals were immunized twice, 8 weeks apart. Recombinant activin A was administered to one group (6 RM) of immunized animals for three consecutive days upon the first and second immunizations (Figure 1A). Env trimer-specific IgG were measured 2 weeks after the booster immunization (week 10) and at the time of necropsy (week 14, 6 weeks post boost). Due to variable background noise detected in the serum of some animals pre-immunization (Supplementary Figure 1), we calculated Env trimer IgG titers as fold change over time. Administration of activin A was associated with a moderate but significant increase in Env trimer IgG titer fold change at week 10 (*p* = 0.04, Figure 1B). Moreover, Env trimer-specific IgG titers were significantly higher in activin A treated animals at 6 weeks post boost (*p* = 0.03, Figure 1B). Interestingly, the treatment with activin A did not result in a significant change of Env V3-loop-specific IgG (Figure 1C), which are “easy to generate” non-neutralizing Abs against the V3 loop tip that becomes inadvertently exposed on non-native Env trimers. The finding of enhanced Env trimer-specific IgG titer fold change was coupled with a trend for higher neutralizing Ab titers at week 10 (*p* = 0.065, Figure 1D). In line with higher Env trimer-binding IgG titers at week 14, activin A-treated animals developed 5-fold more Env trimer-specific IgG secreting cells in bone marrow (Figure 2). Overall, these data suggest that activin A influenced the quality and the persistence of Ab responses to HIV Env trimers in a primate model.

**Figure 3:**
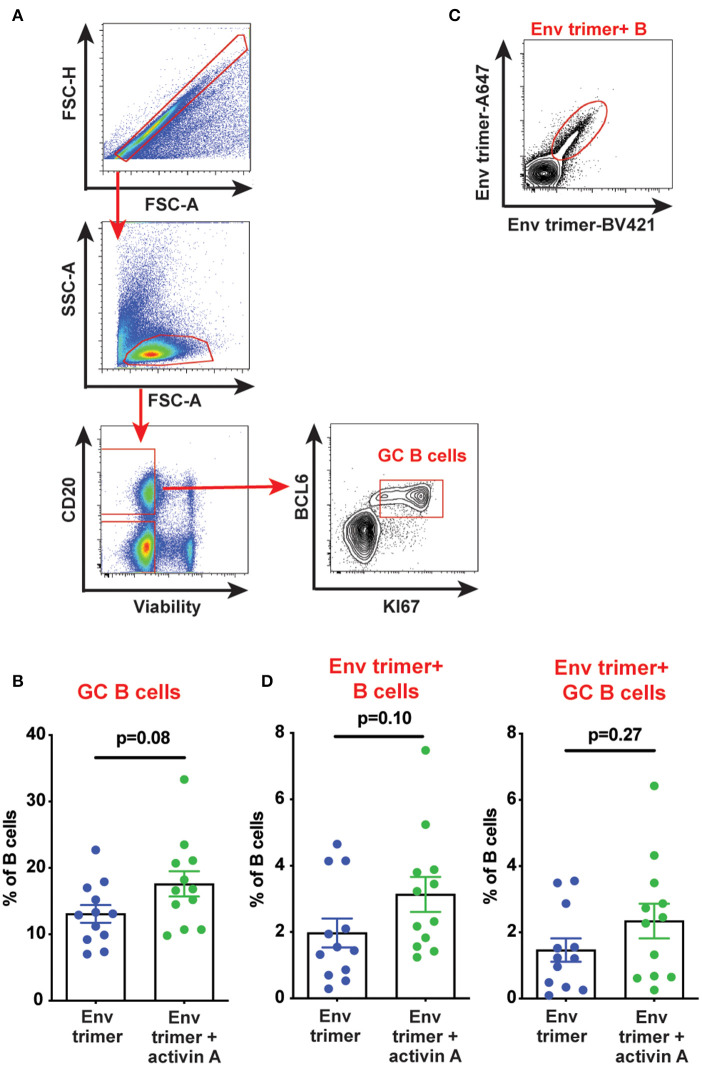
Effect of activin A administration on B cell responses. B cell populations were analyzed by flow cytometry in iliac lymph nodes at week 14. **(A)** Representative flow cytometry analysis of GC B cells. **(B)** Graph shows quantitation of GC B cell frequency as percentage of CD20^+^ live cells. **(C)** Representative flow cytometry staining of Env trimer-specific B cells. **(D)** Graphs show quantitation of Env trimer-specific B cell and GC B cell frequency as percentage of CD20^+^ live cells and BCL6^+^KI-67^+^CD20^+^ live cells, respectively. In **(B,D)** each symbol represents a pool of “right” or “left” iliac LNs from an independent animal. Bars show mean + s.e.m.

**Figure 4:**
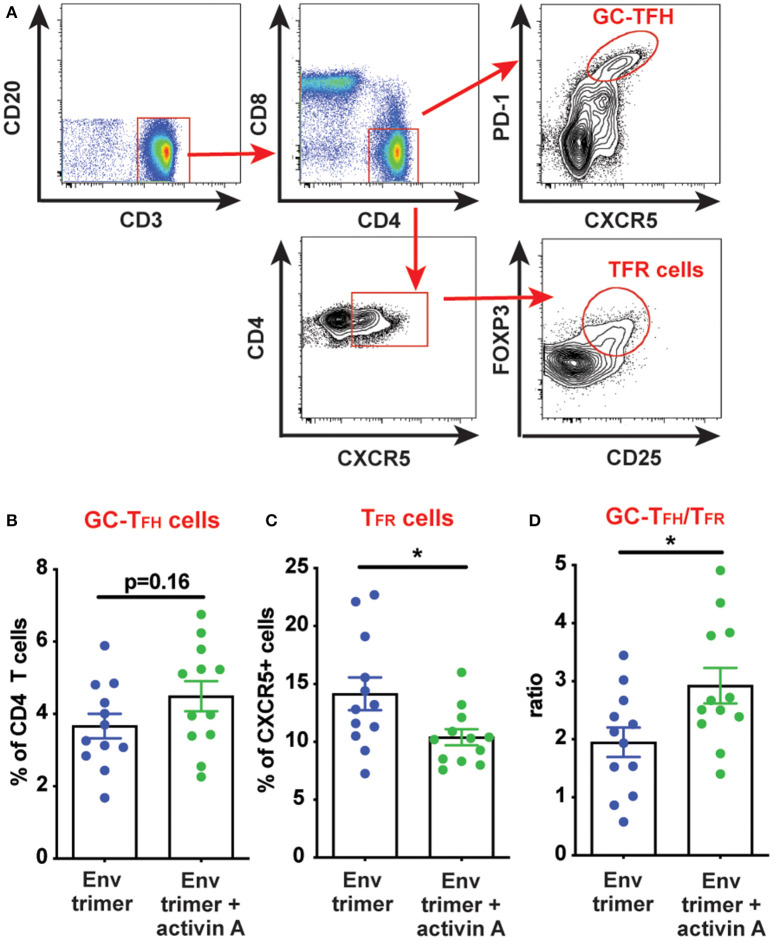
Modulation of T_FH_ and T_FR_ cells by activin A. T cell populations were analyzed by flow cytometry in iliac lymph nodes at week 14. **(A)** A representative gating strategy is depicted for GC-T_FH_ cells and T_FR_ cells. Initial plot is gated on CD20^−^ live cells. **(B,C)** Graphs show quantitation of: **(B)** GC-T_FH_ cell frequency as percentage of CD4^+^CD8^−^CD3^+^CD20^−^ live cells; and **(C)** T_FR_ cell frequency as percentage of CXCR5^+^CD4^+^CD8^−^CD3^+^CD20^−^ live cells. **(D)** Ratio of GC-T_FH_ cells to T_FR_ cells within CXCR5^+^ CD4 T cells at the necropsy time point. In **(B–D)** each symbol represents a pool of “right” or “left” iliac LNs from an independent animal. Bars show mean + s.e.m. *p < 0.05.

We hypothesized that activin A might work as an adjuvant *in vivo* and boost Ab responses by promoting GC B cells and T_FH_ differentiation. Thus, we first measured the frequency of GC B cells in draining iliac lymph nodes (LN) at the necropsy time point (6 weeks post-booster immunization) by flow cytometry (Figure 3A). A non-significant trend was observed for higher GC B cell frequencies in animals previously treated with activin A (*p* = 0.08, Figure 3B). By taking advantage of fluorescently-labeled BG505 Env trimer probes, we monitored the generation of Env trimer-specific B cells and GC B cells (Figure 3C). While no difference reached statistical significance, there was a trend of increased frequency of Env trimer-binding B cells and GC B cells at necropsy in RMs that received activin A (*p* = 0.10, Figure 3D). Next, we assessed the frequency of GC PD-1^hi^CXCR5^+^ GC-T_FH_ cells at the necropsy time point (Figure 4A), and found that GC-T_FH_ cell frequencies were not elevated at this late time point (Figure 4B).

Ab responses can be suppressed by T follicular regulatory (T_FR_) cells. During immune responses, T_FR_ differentiate from T regulatory (T_REG_) cells to acquire several features of T_FH_ cells (CXCR5, BCL6), while lacking B cell helper activity (19, 20). Thus, a vaccine approach capable of promoting T_FH_ cell responses while dampening T_FR_ cells could theoretically elicit superior antigen-specific Ab responses. Given the improved Ab and bone marrow PC responses mediated by activin A, we next sought to determine if activin A administration at the time of immunizations was associated with reduced T_FR_ cell frequencies. T_FR_ cells express the chemokine receptor CXCR5, along with signature molecules of T_REG_ cells such as FOXP3 and CD25 (19, 20) (Figure 4A). Interestingly, activin A treatment dampened T_FR_ frequencies (*p* = 0.03, Figure 4C) and led to significantly increased GC-T_FH_ to T_FR_ ratios (*p* < 0.03, Figure 4D). Altogether, the data generated in this study indicated that activin A can enhance the quality and durability of Env-specific Ab responses, and those outcomes correlated with a favorable bias in the GC-T_FH_ to T_FR_ ratios.

## Discussion

T_FH_ cells are crucial regulators of Ab responses and are necessary for the generation of high affinity LLPC and memory B cells (10, 11). In line with the importance of T_FH_ cells in modulating affinity-matured Ab responses, highly functional blood T_FH_ cells have been found by us and others to correlate with bnAb generation in HIV infected people (21, 22). This finding paved the road to the idea that a vaccine approach capable of fostering T_FH_ differentiation/function could theoretically elicit superior HIV-specific Ab responses (5). Indeed, T_FH_ cells limit the magnitude of GC reactions, and fostering T_FH_ responses might influence the extent of somatic hypermutation as well as the recruitment of rare precursor of nAbs into the GC (5). In light of activin A's capacity to potently shape T_FH_ cell biology *in vitro* (12, 23), we hypothesized that the administration of activin A *in vivo* during immunizations with BG505 SOSIP Env trimers would promote Env trimer-specific Ab responses via modulation of T_FH_ cell biology. Interestingly, activin A administration simultaneously strengthen Env trimer-specific IgG plasma titers as well as bone marrow Env trimer-specific PCs 6 weeks after the booster immunization. Although future experiments with extended evaluation of post-immunization Ab kinetic will be required to fully assess the impact of activin A on Ab longevity, the data observed in this study suggested a role for activin A in regulating the durability of Ab responses by supporting LLPC development. This is a potentially relevant finding, considering that one limitation of current vaccine approaches for HIV is the inadequate persistence of protective Abs (7). An intrinsic adjuvant effect of activin A on T_FH_ cell biology may contribute to the observed improvement of Ab responses, as suggested by the trend in higher T_FH_ cell frequency of activin A treated animals at the necropsy. The increase of T_FH_ cells in response to activin A was moderate and did not reach statistical significance, conceivably because of the time point of analysis. Indeed, our necropsy time point was far from the peak of T_FH_ responses, which usually occurs 7–9 days post immunization in mice (24, 25) and between 2 and 3 weeks post immunization in rhesus macaques when combined with a strong adjuvant (18, 26). Additionally, activin A was last administered 5 weeks before the analysis of T_FH_ cells, and recombinant cytokines have a limited half-life *in vivo*. Thus, it is reasonable to speculate that an extended treatment with activin A or the usage of a strong activin A-inducing adjuvant could result in a more evident persistence of T_FH_ cell at later analysis time points. Activin A might also play a role in the function of T_FH_ cells *in vivo*, thus modulating the quality of Ab responses. Consistent with this scenario, we found enhanced production of BG505 trimer binding Abs and a slight increase of nAbs in RM that were treated with activin A, while V3-specific IgG were unchanged. A recent study from our group demonstrated that slow delivery immunization with Env trimers gives rise to enhanced neutralizing Ab persistence over time (26). Although the underlying mechanism described in this study is the modulation of immunodominance driven by the extended release of Env trimers, it would be intriguing to assess if the prolonged exposure with antigens adjuvanted in soluble ISCOMs-class saponin triggers a sustained *in situ* production of activin A, which in turn contributes to the Env trimer-specific Ab magnitude, quality and persistence.

Another interesting observation that might explain the outcome in the regulation of Env trimer-specific Ab responses is the decreased T_FR_ cell frequency combined with elevated GC-T_FH_ to T_FR_ ratios within CXCR5^+^ CD4 T cells. Although during the course of acute viral infections T_FR_ cells develop at late time points and their main purpose appears to consist in restraining the formation of autoreactive Ab secreting cells (25), multiple papers have shown that upon immunization with certain protein antigen-adjuvant combinations T_FR_ cells can suppress GC reactions and the production of Ag-specific Abs (27–29). Interestingly, it was suggested in some studies that the proportion of T_FH_ relative to T_FR_ cells might be used as proxy for predicting the magnitude of GC responses and antigen-specific Ab generation (30–32). The skew in favor of GC-T_FH_ cells that we observed in our study could result from: (1) a direct effect of activin A in promoting T_FH_ differentiation (as discussed above); (2) an inhibitory role of activin A in T_FR_ differentiation/maintenance; or (3) a combination of these two mechanisms. While no study directly assessed the effect of activin A in T_FR_ differentiation from T_REG_ cells, the analysis of publicly available transcriptome data from monkeys (31) and mice (33) revealed a detectable expression of activin A receptors on T_REG_ cells. Hence, local activin A concentrations might be sensed by T_REG_ cells and influence their differentiation toward T_FR_ *in vivo*. Since we previously described that activin A drives a strong expression of PD-1 in naïve CD4 T cells *in vitro*, we speculate that a similar induction of PD-1 might divert T_REG_ cells from T_FR_ differentiation. Indeed, PD-1 has previously been shown to restrain T_FR_ cell differentiation in mice (29). Future studies will be required to further address the direct effect of activin A on T_FH_ and T_FR_ cell biology *in vivo*, and might be able to shed light on the mechanisms required for the generation of protective, long-lasting Ab responses against pathogens difficult to neutralize, such as HIV.

In sum, our study highlights the beneficial activity of activin A in promoting antibody responses *in vivo* in the context of vaccination. Since recombinant cytokines are not suitable to be exploited as adjuvants in commercial vaccine formulations due to production cost and stability issues, we suggest that increasing efforts should be directed at characterizing and potentially pursuing activin A-inducing adjuvants for future vaccine rational design with the goal of fostering superior humoral responses to vaccines.

## Data Availability Statement

All datasets generated for this study are included in the article/Supplementary Material.

## Ethics Statement

This study was approved by the Emory University Institutional Animal Care and Use Committee (IACUC).

## Author Contributions

ML, DC, KK, AE, CE, EG, PD, and MP performed experiments and/or analyzed data. GO and AW generated BG505 SOSIPv5.2 HIV Env trimer protein. MR, RA, DB, and GS contributed to scientific discussion and manuscript editing. ML and SC designed the study and wrote the manuscript. All authors contributed to the article and approved the submitted version.

## Conflict of Interest

ML and SC filed a patent application PCT/US15/63500 Modulators of activin and methods for modulating immune responses and T follicular helper cells. The remaining authors declare that the research was conducted in the absence of any commercial or financial relationships that could be construed as a potential conflict of interest.

## Supplementary Material

Supplementary Figure 1 HIV-specific Ab regulation by activin A. **(A,B)** Env trimer **(A)** and Env-V3 **(B)**-specific IgG titers before immunization (preimmune) and at week 10 and week 14 post immunization are shown. Bars show geometric mean with geometric SD. ^**^*p* < 0.01.

Supplementary Table 1 List of animals' sex; weight, age, previous treatment status at beginning of the study; and immunization and treatment received during the study.
