# Supplementary figures and images for: Harnessing Activin A Adjuvanticity to Promote Antibody Responses to BG505 HIV Envelope Trimers
Source: Front Immunol. 2020 Jun 16;11:1213. doi: 10.3389/fimmu.2020.01213 (PMC7308430; doi:10.3389/fimmu.2020.01213)

Figure S1

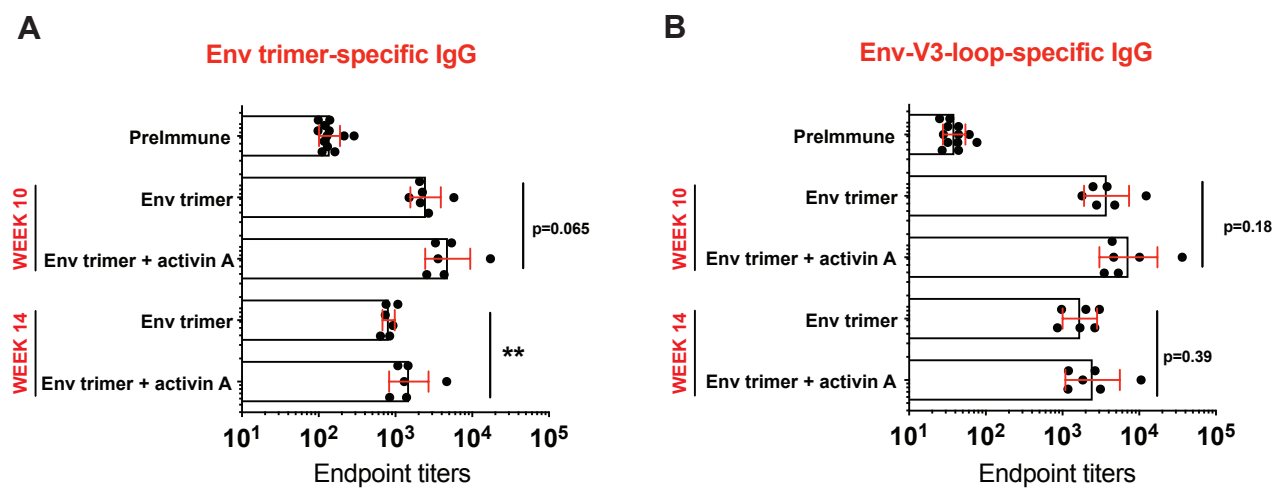

Supplement: Supplementary Figure 1 — HIV-specific Ab regulation by activin A. (A,B) Env trimer (A) and Env-V3 (B)-specific IgG titers before immunization (preimmune) and at week 10 and week 14 post immunization are shown. Bars show geometric mean with geometric SD. **p < 0.01. [file Data_Sheet_1.PDF]
