# Supplementary material for: Harnessing Activin A Adjuvanticity to Promote Antibody Responses to BG505 HIV Envelope Trimers
Source: Front Immunol. 2020 Jun 16;11:1213. doi: 10.3389/fimmu.2020.01213 (PMC7308430; doi:10.3389/fimmu.2020.01213)
Supplement: Supplementary Table 1 — List of animals' sex; weight, age, previous treatment status at beginning of the study; and immunization and treatment received during the study. [file Data_Sheet_2.pdf]

Supplementary Table 1

Animal	Sex	Weight (kg) start of study	Age start of study	Previous treatment	Immunization	On study treatment
RYf16	Male	5.2	2 yr 10 mo	None	Env trimer + ISCOMATRIX	Activin A
RWf16	Male	4.9	2 yr 10 mo	None	Env trimer + ISCOMATRIX	Activin A
RVa16	Male	4.7	3 yrs	None	Env trimer + ISCOMATRIX	Activin A
RFo15	Male	6.09	3 yr 11 mo	None	Env trimer + ISCOMATRIX	Activin A
RBd15	Male	6.31	4 yr 1 mo	None	Env trimer + ISCOMATRIX	Activin A
RWr15	Male	6.26	4 yr 1 mo	None	Env trimer + ISCOMATRIX	Activin A
RWi15	Male	8.1	4 yr	None	Env trimer + ISCOMATRIX	None
RHf15	Male	8.15	4 yr	None	Env trimer + ISCOMATRIX	None
RRe16	Male	4.4	2 yr 11 mo	None	Env trimer + ISCOMATRIX	None
RUe16	Male	5.52	2 yr 11 mo	None	Env trimer + ISCOMATRIX	None
RQc16	Male	5.36	3 yr	None	Env trimer + ISCOMATRIX	None
REu15	Male	4.9	2 yr 10 mo	None	Env trimer + ISCOMATRIX	None
